# Methodologies for Fabricating Flexible Supercapacitors

**DOI:** 10.3390/mi12020163

**Published:** 2021-02-07

**Authors:** Seohyeon Jang, Jihyeon Kang, Soyul Kwak, Myeong-Lok Seol, M. Meyyappan, Inho Nam

**Affiliations:** 1 School of Chemical Engineering and Materials Science, Department of Intelligent Energy and Industry, Institute of Energy Converting Soft Materials, Chung-Ang University, Seoul 06974, Korea; tjgus6142@cau.ac.kr (S.J.); kar04114@cau.ac.kr (J.K.); chatte0614@cau.ac.kr (S.K.); 2 Center for Nanotechnology, NASA Ames Research Center, Moffett Field, CA 94035, USA; myeonglok.seol@nasa.gov (M.-L.S.); meyya@ieee.org (M.M.); 3 Universities Space Research Association, NASA Ames Research Center, Moffett Field, CA 94035, USA

**Keywords:** supercapacitors, flexible electronics, wearable devices, micro-supercapacitors

## Abstract

The spread of wearable and flexible electronics devices has been accelerating in recent years for a wide range of applications. Development of an appropriate flexible power source to operate these flexible devices is a key challenge. Supercapacitors are attractive for powering portable lightweight consumer devices due to their long cycle stability, fast charge-discharge cycle, outstanding power density, wide operating temperatures and safety. Much effort has been devoted to ensure high mechanical and electrochemical stability upon bending, folding or stretching and to develop flexible electrodes, substrates and overall geometrically-flexible structures. Supercapacitors have attracted considerable attention and shown many applications on various scales. In this review, we focus on flexible structural design under six categories: paper-like, textile-like, wire-like, origami, biomimetics based design and micro-supercapacitors. Finally, we present our perspective of flexible supercapacitors and emphasize current technical difficulties to stimulate further research.

## 1. Introduction

Wearable and flexible electronics has been gaining much attention recently due to the potential of the emerging *Internet of Things* (IOT) and related applications such as smartwatches, head mounted-displays, Bluetooth-earphones, smart clothing, foldable mobile phones, electronic skins and medical equipment. These applications have made the distance between humans and devices smaller, resulting in a whole new set of performance metrics and expectations—in addition to the usual metrics in each case—that include endurance under unusual conditions of bending, stretching, exposure to ambient including water and others. Advances in flexible sensors [1], transistors [2,3,4], displays [5,6] and touch screens [7] have been reported in recent years to meet application needs. Development of an optimal and appropriate power source in terms of energy/power density, fast charging and footprint is a challenge facing these applications right now. Conventional energy storage systems (ESS) such as the rigid supercapacitors (SC) and Li-ion batteries (LiB) are not suitable because of their heavy weight relative to the application scenario, hardness and bulk size. For example, lithium-ion coin-type batteries have powered simple fitness band style sensors, but they face serious limitations with the introduction of more complex smartwatches capable of multiple functions. The requirements to power these portable and flexible cutting-edge electronic devices are as follows: First, the electronic components must be miniaturized sufficiently to accommodate all the desirable features and the power source must match the application device footprint. Second, the power source must be light and immune from explosion. Third, the life of the power source must extend to long-term use on a single charge, as most applications demand continuous signal detection from the body or outside. Last, the power source must be ideally deformable as demanded by the application while maintaining its electrochemical performance. Flexible SCs meet most of the desirable characteristics—as will be seen in this review—such as ultrafast charging, high power density, good storage capacity and high expandability without significant performance degradation. Further, its long cycle life, wide operating temperatures and safety range make it more suitable than conventional batteries [8,9,10,11,12]. Here, we provide a review of the most recent and promising examples of flexible SCs, classified by fabrication methodologies.

## 2. Design Strategies for Flexible Supercapacitors

Improving the flexibility along with a higher level of compact design of flexible SCs requires innovations in the structural design of the electrode materials [13]. The basis is a conductive and flexible substrate that can be used as a current collector, in addition to flexible electrodes with high electrical conductivity for fast charging and discharging. Recent research developments in this aspect can be grouped under the following categories: paper and paper-like, textile and wire-shape configurations [14]. Paper and textile are recognized as optimal substrates owing to their low cost, flexibility and highly porous structure capable of absorbing active electrode materials. The wire-shaped SCs are small in size, lightweight, highly flexible and can be transformed into any shape. This section introduces all three categories and reviews their relative strengths and weaknesses in meeting the common and basic criteria for flexible SCs.

### 2.1. Paper and Other Paper-Thin Substrates

**Figure 1:**
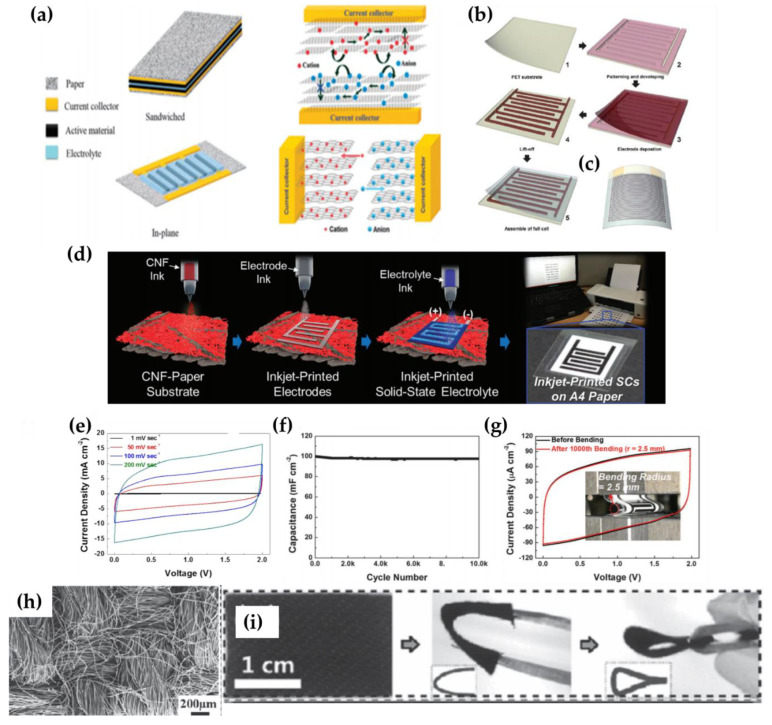
(**a**) Illustration of paper type SCs with a traditional “sandwich” structure and a new “in-plane” structure. Diagram exhibiting the ion transfer associated with the operation of both types of supercapacitors (SCs). Adapted with permission from [17]. Copyright (2015), The Royal Society of Chemistry 2015. (**b**) Process flow for producing transparent, super-bendable supercapacitor without percolation. (**c**) Schematic of a transparent, ultra-bendable supercapacitor with alternating patterned electrodes. Adapted with permission from [17]. Copyright (2013), The Royal Society of Chemistry 2013. (**d**) Manufacturing of inkjet-printed SCs with different fabrication factors. Schematic diagram of the stepwise manufacturing procedure of an inkjet-printed SC. (**e**) Cyclic voltammetry (CV) profiles at various scan rates (1–200 mV/s). (**f**) Cycling performance of inkjet-printed SC at a constant charging-discharging current density (0.2 mA/cm^2^). (**g**) CV tests of inkjet-printed SC (scan rate of 1.0 mV/s) after repetitive folding deformation (folding radius from 2.5 mm to 1000 cycles) [22]. Copyright (2016), The Royal Society of Chemistry 2016. (**h**) SEM image of activated carbon fiber cloth (ACFC). (**i**) ACFC based textiles are bent with a plastic tube of diameter 8.5 mm. Adapted with permission from [31]. Copyright (2015), WILEY-VCH Verlag GmbH & Co. KGaA, Weinheim.

Paper is a promising substrate for constructing flexible energy storage devices due to its large surface area and mechanical strength. It serves as excellent support for loading active materials and electrolytes and has features that can improve the life cycle with high power density and energy density relative to conventional rigid electrodes when physical stress is applied [15]. The two main electrode designs in paper SCs are the “sandwich” and the “in-plane” devices as shown in Figure 1a [16]. Yuan et al., fabricated sandwich-type supercapacitors using a carbon nanoparticle (CNP)/MnO_2_ nano-rod hybrid design and H_3_PO_4_/polyvinyl alcohol (PVA) electrolyte [12]. The supercapacitors fabricated on paper-thin carbon fabric were lightweight, flexible and twisted without compromising the structurally integrated devices. The cyclic voltammetry (CV) results showed only subtle changes in electrochemical performance at various bending angles and retention of 97.3% of its initial capacity even after 10,000 charge-discharge cycles [12].

While almost every paper type SC uses conventional sandwich electrodes, this design cannot compete with new configurations based on in-plane interdigitated electrodes that feature higher power and energy densities [16]. Nam et al., fabricated transparent and ultra-bendable supercapacitors with in-plane interdigitated electrodes using a masking method (Figure 1b) [17]. They deposited Au and active materials (MnO_2_) on polyethylene terephthalate (PET) and assembled transparent PVA/H_3_PO_4_ gel polymer electrolyte at the last stage. This paper-thin supercapacitor displayed superior capacitance stability under in-plane bending and compressive conditions [17]. The capacitance increased by ~1.2 times when a flat supercapacitor was curved with the internal highest bending rate covering the transparent supercapacitor. Then, the compression bending of the electrolyte caused pressure in the direction perpendicular to the PET substrate in the PVA/H_3_PO_4_ electrolyte membrane, resulting in a closer interaction between the electrode and electrolyte membrane [18,19,20,21].

The interdigitated electrode pattern can be made using masking or by direct printing. The example above used the masking approach. Printing enables mass production of a thin digital design pattern since the inks are amenable to producing ultra-thin patterns on pre-engineered substrates. The printing process begins by dispersing inorganic nanoparticles (NPs) or organic dye in a proper solvent to develop an ink of appropriate viscosity. Printing technology is particularly well suited for the manufacture of flexible, low-cost, portable products because of the generality and broad applicability of substrates and inks. Choi et al., demonstrated inkjet printing on paper utilizing a common desktop inkjet printer [22] and Figure 1d is a schematic representation of the step-by-step procedure using the desktop printer. Figure 1e shows the CV profile for the fabricated device that exhibits an almost rectangular shape at various scan rates from 1 to 200 mV/s. The cycling performance of the SC was tested at a constant charging-discharging current density (0.2 mA/cm^2^) and there was no significant decrease in the cell capacitance (about 100 mF/cm^2^) over 10,000 charge/discharge cycles (Figure 1f). The SCs also maintained their structural shape after 1000 bending deformations without degrading the capacitance of the cell (Figure 1g).

The layer-by-layer (LbL) assembly is an easy way to accurately control the number of active materials loaded on diverse substrates based on interdependent interactions between species, regardless of the size and shape of the substrate [23,24]. Ko et al., fabricated bendable paper-like SCs using LbL assembly based on hybrid asymmetric structural composition [25]. Au-soaked paper was used as substrate with MnO cathode, Fe_3_O_4_ anode, PVA/Na_2_SO_4_ polymer gel electrolyte and a separator. The contact resistance between adjacent NPs was minimized to increase the areal capacitance and rate performance by directly bridging all the interfaces of either metal, metal oxide nanoparticles, or both, through a small TREN ligand (tris(2-aminoethyl)amine). The assembled SC showed a specific power density of 128.9 kW/kg, specific energy density of 121.5 W h/kg and energy density value of 267.3 μW h/cm^2^. The device exhibited an area power value of 15.1 mW/cm^2^ and areal capacitance of 1.35 mF/cm^2^ at a high NP loading amount of >4.09 mg/cm^2^, and about 90% of the initial capacitance was retained after 5000 cycles. The SC also exhibited outstanding mechanical stability under various stress conditions, which is crucial for practical implementation. There was no significant change in the CV shape during bending or wrapping, indicating a solid and stable connection between the paper substrate, NP and electrolyte [25].

### 2.2. Textile Configuration

Carbon fiber is an up-and-coming candidate for flexible substrates due to its high mechanical strength and electrical conductivity. The textile’s 3D network can provide fast electron and ion conduction paths and high loading quantity of active materials. The textile scaffolding can be fabricated from carbon nanotubes (CNT), graphene fiber, metal, et cetera [26,27,28,29,30,31]. Dong et al., chose activated carbon fiber cloth (ACFC) as the body material to design ACFC/CNT and ACFC/MnO_2_/CNT composites [31]. The ACFC/MnO_2_/CNT textile electrode has a long operating life and excellent flexibility as fiber and textile electrodes. The manufactured textile electrode showed an areal capacitance of 2542 mF/cm^2^, power density of 16,287 μW/cm^2^ and energy density of 56.9 μWh/cm^2^. Textile and fiber electrodes provide excellent cycling performance and structural flexibility. Figure 1h shows an SEM image of ACFC, which is good for constructing electrochemical double-layer capacitor (EDLC) electrodes [28,29,30]. The ACFC textile was woven from activated carbon fiber bundles (ACFBs), which were fabricated from thousands of twisted activated carbon fibers. Mechanically strong and flexible carbon fiber provides constant strength and excellent flexibility for ACFC-based textiles and ACFB-based fiber bundles. The composite textile can be bent or rolled into a plastic tube as seen in Figure 1i, and the composite fiber bundle can be distorted, knotted or woven into a simple fabric like frame [31].

Cheng et al., produced textile electrodes woven from CNT/graphene fibers (GF) which have high electrical conductivity and surface area by pre-intercalating Fe_3_O_4_ nanoparticles [32]. 

The CNT/GFs retain outstanding flexibility of the GFs by bending them into loops or springs without structural breaks. The folding state of the textile supercapacitor exhibited a CV curve similar to that when in the flat state. The capacitance decreased initially during cycle tests but leveled out at a stable value after 1000 cycles (0.4 mF/cm^2^); the box-shaped CV curve also slowly contracted and stabilized over 200 folding cycles [32].

Cakici et al., reported highly flexible carbon-based textile well covered with MnO_2_ structures [33]. Owing to its 1D construction, carbon textiles are the most commonly used current collectors in energy storage applications. MnO_2_ can be grown directly on the surface of carbon textile collectors with a horizontal 1D structure and thus can produce a supercapacitor electrode without using a conductive additive or a binder. The charge and discharge capacitance remained at the initial value of 461 F/g after 5000 cycles, and the capacitance maintenance ratio of the carbon textile-MnO_2_ hybrid device was 99.7%. Furthermore, the Coulombic efficiency was maintained at 99.3%, indicating the stability of the device. The fabricated composite electrode has indicated a specific capacitance of 463 F/g at 1 A/g in 1.0 M Na_2_SO_4_ electrolyte and good cycling stability by maintaining excellent capacitance at high C-rate [33].

### 2.3. Wire Configuration

**Figure 2:**
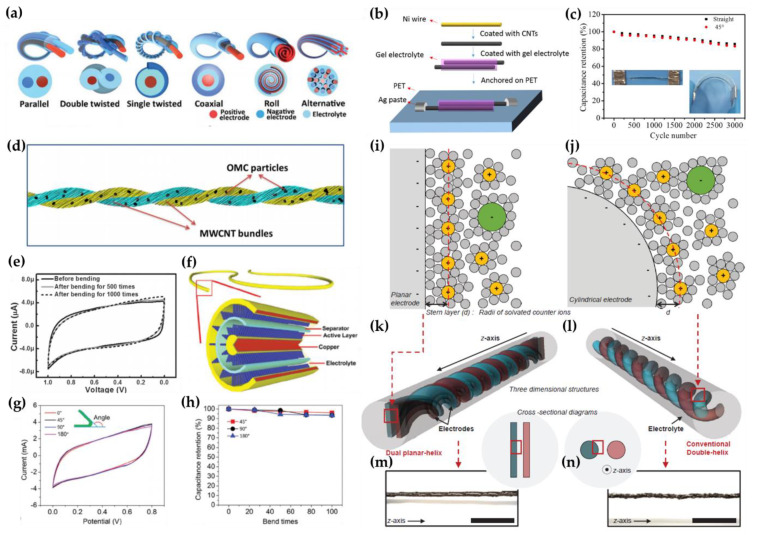
(**a**) Main structures and cross-sections of various wire type supercapacitors [40]. Copyright (2021), Materials Chemistry Frontiers. (**b**) Description of the structure and schematic of the fabrication procedure for a flexible spiral supercapacitor. (**c**) Cyclic stability of wire supercapacitor before and after 45-degree bending during charging-discharging at current density 0.8 A/g. The insets indicate the wire supercapacitors in their original state (left) and their bent state (right) [37]. Copyright (2018), Advanced Materials. (**d**) Schematic of an electrochemical double-layer capacitor (EDLC) wire composed of two multi-walled carbon nanotube/ordered mesoporous carbon (MWCNT/OMC) composite fibers. (**e**) CV curves of EDLC wires (OMC weight percent 87%) before and after 500 and 1000 cycles of bending [35]. Copyright (2013), Advanced Materials. (**f**) Schematic illustrations of coaxial supercapacitor cable with solid electrolyte. (**g**) CV graphs (at a scan rate of 100 mV/s) for various bending angles in the range from 0 to 180°. (**h**) Folding the device up to 100 times at various bending angles to show superior bendability of the device. Adapted with permission from [36]. Copyright (2014), WILEY-VCH Verlag GmbH & Co. KGaA, Weinheim. (**i**) Schematic of electric double layer on a planar electrode. (**j**) Cylindrical electrode structure of (**k**) dual planar-helix and (**l**) double helix wire type SCs. (**m**) Dual planar-helix and (**n**) double helix wire type optical images [46]. Copyright (2016,) Advanced Energy Materials.

Unlike the traditional paper type flexible SCs, the recently introduced wire type SCs are smaller in size, lighter in weight, more flexible and can be transformed into almost any shape, knotted and even woven into textiles. Structural type is crucial for the device assembly and operational stability in wire type SCs. Three structural designs have been developed to date: parallel [34], twisted [35] and coaxial [36] configurations, as shown in Figure 2a. Li et al., fabricated wire-shaped supercapacitor electrodes built through growing CuCo_2_O_4_ nanostructures onto Ni wires [37]. In general, it is hard to control the morphology of the grown nanostructure. To deal with these issues, they induced facile capillary action to assemble the single-walled CNTs (SWCNTs) and graphene oxide (GO) directly on the nickel wire (Figure 2b). The adsorption of CNTs on parallel Ni surfaces is facilitated by the shape of the nanowires, which significantly enhances conductivity and promotes electrolyte penetration. These symmetrical all-solid wire-shaped SCs exhibit outstanding EDLC performance in addition to ultrahigh flexibility and mechanical properties. They attained 34.7 F/g of specific capacitance which persisted at 83% of initial value over 3000 cycles of bending and relaxing by 45 degrees as shown in Figure 2c [37].

The two fiber electrodes in a parallel structure are physically separated from each other and can become mechanically unstable. Therefore, Ren et al., reported a flexible and wearable EDLC wire by twisting two aligned multi-walled carbon nanotube (MWCNT)/ordered electrodes (Figure 2d) [35]. Figure 2e exhibits no apparent degradation in electrochemical performance when the EDLC is bent. The ordered mesoporous carbon (OMC) particles are tightly bundled by aligned MWCNTs, allowing more effective use of the high surface area of OMC components. The CV curve of the EDLC wire was well maintained at a scan rate of 1 mV/s during the 1000 cycle bending process [35]. Parallel and twisted fibers placed in the center of the devices exhibit the shortest distance, while the fibers located on the outside of the devices show the maximum distance. The longer the distance between the cathode and anode, the longer the ion diffusion path, which results in higher internal resistance and lower power density [38,39].

Owing to the relatively low ion mobility of gel electrolytes compared to liquid electrolytes, it is necessary to optimize the distance between the fiber electrodes to improve the overall performance of the flexible device. On the other hand, the coaxial type demonstrates a more homogeneous distance between electrodes and shows efficient charge/ion transfer [40]. In addition to improving the charge/ion transfer, the coaxial type is regarded as a more mechanically stable configuration [41,42]. This design also allows the flexibility to merge two different devices into one device to perform the original function.

Yu et al., fabricated freestanding CuO@AuPd@MnO_2_ SCs using coaxial nano-whiskers (NWs) [36]. Figure 2f shows an illustration of the coaxial supercapacitor cable (CSC) using a solid electrolyte (SE). Thin AuPd was deposited onto the CuO NWs to act as a current collector and the electrodeposited MnO_2_ acted as the anode. It can be easily fabricated by placing the outer tubular electrode (both pre-coated with gel electrolyte) over the two electrodes partitioned by a separator which has ionic porosity. Built from these NWs, this supercapacitor showed outstanding bendability and flexibility, high energy density, high power and excellent cyclic stability. Figure 2g indicates the shape of CV curves to be the same for folding angles from 0 to 180 degrees. In addition, the box and symmetrically shaped CV curves show the ideal pseudocapacitive property of MnO_2_ and excellent reversible oxidation-reduction reaction. This device maintained 93.4% of its initial capacitance after bending 100 times at 180 degrees (Figure 2h), showing considerable bendability [36]. However, it is hard to accurately assemble a multi-layered core-sheath design into long fibers with a small diameter. Consequently, it is necessary to develop a simple process for producing new configurations that can keep a constant distance between the electrodes [43,44]. Nam et al., called this difficulty an “energy lag effect” [45]. When two types of electrodes are formed between the planar and cylindrical electrode, the electric field is different. For example, the electric field in a normal charging plane is presumed to be homogeneous in the 1D direction. In contrast, the electrodes of a wire-shaped energy storage device have a cylindrical structure, and in reality, an electric field that attenuates in 2D is generated. To avoid the effects of this energy lag effect, Nam et al., proposed a dual planar-helix structure for the electrodes, which has an entire wire type but the capacity is analytically equivalent to that of a normal 2D planar SC. The electro-capacity and the ohmic resistance of the planar and double-helix designs were investigated using CV and galvanostatic charging-discharging (GCD). The capacitance density of a dual planar-helix supercapacitor (1.9 F/cm^3^ at 10 mV/s) was found to be three times higher than that of a double helix type (0.66 F/cm^3^ at 10 mV/s) made using the same materials. Furthermore, the CV curves showed stable electrochemical performance under twisting deformation [45].

**Figure 3:**
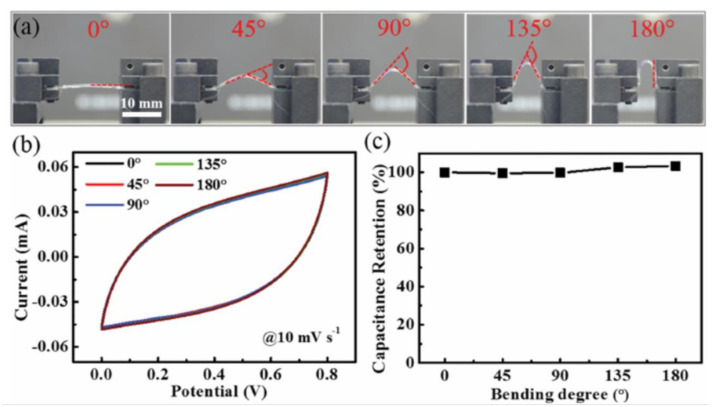
(**a**) Digital illustrations of a parallel double helix structure (PDHS) in different bent states. (**b**) Corresponding CV graphs and (**c**) the capacitive retention property at different bent states. Adapted with permission from [47]. Copyright (2016), WILEY-VCH Verlag GmbH & Co. KGaA, Weinheim.

Guo et al., developed a wire-type supercapacitor with a parallel double helix structure (PDHS) for stable operation even under deformed states [47]. They wrapped two symmetrical titanium @MnO_2_ (Ti@MnO_2_) fiber electrodes around flexible nylon fiber and separated them by a spatial gap filled with LiCl/PVA gel as the electrolyte. A commercially available metal Ti fiber with high conductivity was used as current collector and MnO_2_ was electrochemically deposited as active material for the cathode and anode. Figure 3a depicts the mechanical and electrochemical stability of PDHSs at different angles (0–180°). The CV curves (at a scan rate of 10 mV s^−1^) in the bent state are almost the same as shown in Figure 3b. The capacitance change is less than 1% for bends from 0 to 180° (Figure 3c). This structure allows two parallel, twisted fiber electrodes to remain physically separated during bending, ensuring mechanical stability [47].

### 2.4. Origami-Shaped SCs

**Figure 4:**
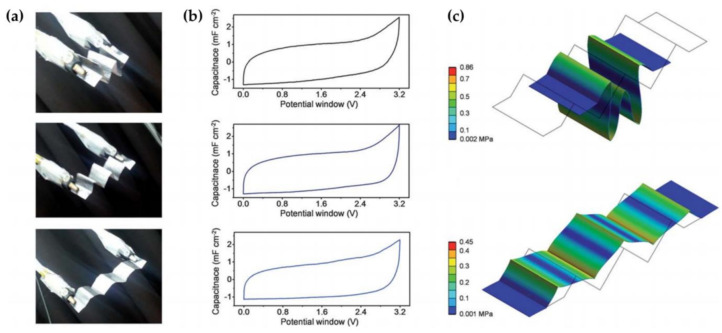
(**a**) Photographs of an origami-based stretchable energy system with three isolated electrodes (IEs) at about 60% (compressible), tensile of 0% and 30%. (**b**) CVs of a compressible state at about 60%, tensile of 0% and 30% (**c**) 3D finite element (3D FEM) modeling of stress distribution for the equivalent system under the same compressibility and tensile strain [49]. Copyright (2014), The Royal Society of Chemistry.

The origami-based approach represents another alternative that allows deformability compared to conventional methods using freely deformable materials and mechanically designed structures. Origami is the ancient art of folding 2D sheets of paper with advanced folding along predefined creases and can be used to fabricate flexible and compact 3D structures [48]. Nam et al., fabricated an origami-based, all-solid-state, bendable supercapacitor system, assembling the analog of a series circuit [49]. This supercapacitor consisted of periodically isolated electrodes (IEs) and sectionalized electrolytes. These are important elements of a single system for the densely packed series circuit analogs. The sectionalized electrolytes and IEs were produced by easily designing with a graphite rod onto a paper substrate. The sectionalized ion transferring paper (SITP) substrate exhibits stable folding characteristics that are natural for ordinary paper. The characteristics of SITP allow origami construction. As shown in Figure 4a, Nam et al., produced three IE samples featuring seven bent in layers with a wavy pattern and confirmed their electrochemical properties. The results indicate a close CV graph under compression (60%), planar and tensile deformation (30%) conditions with specific capacitances of 0.94, 0.98 and 0.93 mF/cm^2^ according to each deformable state in Figure 4b. Furthermore, they demonstrated the numerical analysis of stress distribution through modeling to represent the mechanical properties of each sample (Figure 4c) [49].

### 2.5. Biomimetic Configuration

Nature is a collection of technologies that have long been stabilized, optimized and made efficient and sustainable through the process of evolution. The collaboration between material scientists and biologists is important, namely, in the development of biomimetics, which refers to the imitation and application of systems and mechanisms as well as the structure and functions of organisms adapted to the environment through evolution. It seeks to develop stable and energy-efficient technologies by utilizing the structural features of living things such as multifunctionality, adaptability, resilience and self-organization capabilities. Examples of biomimetics range from materials to robotics [50,51], human organs and tissue development [52,53,54] and power supplies [46,55].

#### 2.5.1. Actin-Myosin Induced Omni-Directional Stretchable System

**Figure 5:**
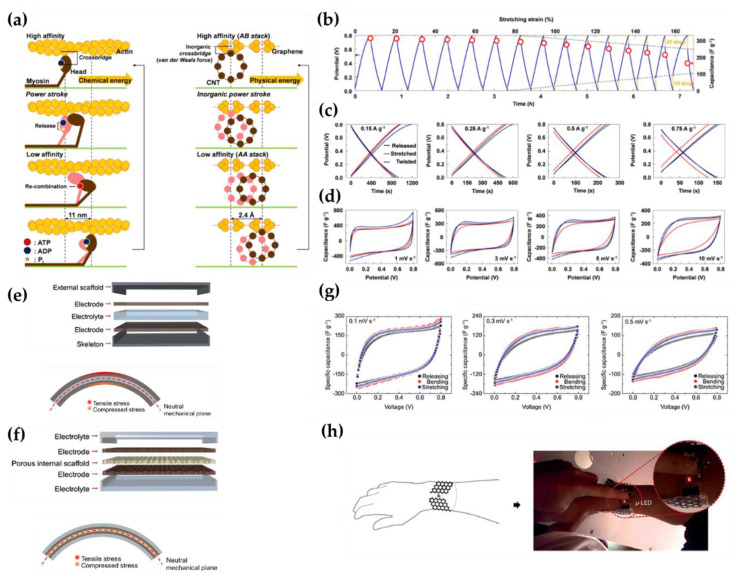
(**a**) Paradigms for the gradual motion of carbon nanotubes (CNTs) along with graphene in an inorganic system and of myosin along actin in a living cell. (**b**) The galvanostatic charging-discharging (GCD) curve for the graphene/CNT layers on polyvinyl alcohol (PVA)/H_3_PO_4_ film at a constant current (0.15 A/g) while stretching the structure at a steady rate of 0.4% strain per minute. (**c**) Representative GCDs at several current densities (from 0.15 to 0.75 A/g) without strain and with twisting (360°) and stretching (80%). (**d**) Representative CVs at diverse scan rates (from 1 to 10 mV/s) without strain and with twisting (360°) and stretching (80%) [55]. Copyright (2017), Biomaterials. (**e**) Bending deformation states of energy storages and schematic drawing of an exoskeleton energy storage system. (**f**) Endoskeleton energy storage system. Stretching features of endoskeletons with hexagonally located pore structures and electrochemical properties of the device. (**g**) Representative CVs at 0.1, 0.3 and 0.5 mV/s. (**h**) Employment of the endoskeleton system in a micro-LED device on a wrist [46]. Copyright (2016), Journal of Materials Chemistry.

The sarcomeres in muscle tissue are composed of myosin molecules and actin filaments. Myosin molecules act as wheels and actin filaments function as tracks and they undergo permanent and reversible stretching in living bodies. Nam et al., imitated the structure of sarcomere to investigate complete and independent stretchable all-solid-state SCs [55]. In this system, graphene-CNT layer and PVA/H_3_PO_4_ were used as electrodes and gel electrolyte, respectively. CNT adapts as a roll of myosin and graphene acts like a floated track (actin filaments). CNT turns into a stretching motion when it receives external stress because of its high elasticity, and by connecting graphene and CNT with van der Waals interaction, the interfacial stress and slip stress are diminished at various deformed states. Figure 5a demonstrates the systematic structure of the electrode. The graphene/CNT-layered structures demonstrate highly stable electrochemical performance under twisting and biaxial and uniaxial transformation. The performance under stretched and twisted conditions was measured by the charge/discharge method and the CV curves of the SC with graphene/CNT electrodes are shown in Figure 5b–d. A higher specific capacitance (329 F/g) is seen compared to the capacitance of traditional electrodes having resistance against a deformed state. The representative values of CD and CV is shown at various currents and scan rates upon releasing and 80% stretching state in Figure 5c,d. It indicates the stable and high energy storage property (349 F/g) at a twisting 360° angle [56,57,58,59].

#### 2.5.2. Endoskeleton Structure Energy Storage System

Regardless of how stretchable and foldable electrodes are developed, fabrication of fully flexible electrical devices is not possible as long as an external hard passive cover exists. The passive cover, electrodes and electrolytes are loaded from the outside to the inside in conventional systems, which have a structure similar to exoskeleton systems like insects. The hard-cover provides stability against physical attacks but limits flexibility. To circumvent this intrinsic problem, Nam et al., proposed an oppositely ordered structure, in other words, endoskeleton structure as shown in Figure 5e,f. They used graphene-CNT layer electrodes for stretchable electrodes and PVA/H_3_PO_4_ as electrolyte because PVA has enough tensile yield strength (23 MPa) and stretchability for use as an external layer [60]. Polypropylene sheets were used as a porous and internal scaffold, which played the roles of a skeleton and ion transferring substrate. The pores are arranged hexagonally to minimize strain and stress while deformed, resulting in serpentine networks. These endoskeleton structured SCs showed great capacitance stability under folded and stretched states. The specific capacitance assessments according to scan rates of 0.1, 0.3 and 0.5 mV/s were 144, 95, and 73 F/g under 15% stretched state (Figure 5g) and the achieved Coulombic efficiency was about 90% regardless of the deformation states. Furthermore, the specific capacitance values were maintained at over 97% and 90% after 50 cycles of bending and stretching, showing steady electrochemical and mechanical performance. Figure 5h represents a simple application of devices for use on the wrist [46].

### 2.6. Micro-Supercapacitors

With an industrial focus on miniaturized autonomous electrical devices, reducing the thickness and size enough to be carried has commanded attention, along with flexibility to easily integrate them into circuits of micro-devices. All-solid-state micro-supercapacitors (MSCs) are especially encouraging to meet the aforementioned purposes [61,62]. However, ions of the electrolyte in conventional electronic devices with piled structures hardly penetrate deep inside the active electrode materials, leading to low C-rates accompanied by comparatively low power and energy densities [63,64,65]. Hence, in-plane MSCs using interdigitated structures were developed to provide high power density because of their short ion diffusion length and densely packed electrodes [66]. Diverse patterning approaches have been established to prepare interdigitated electrodes for MSCs including photolithography with laser scribing [67,68,69], etching [70,71,72] and printing [73,74,75].

**Figure 6:**
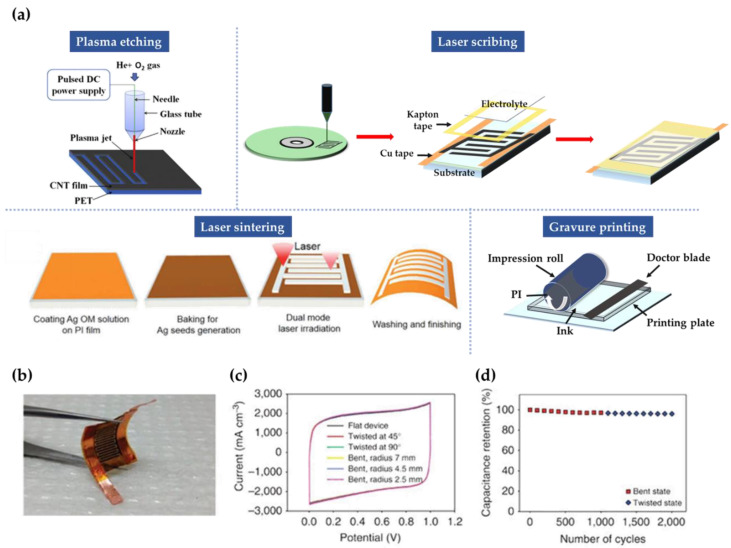
(**a**) Schematic illustration of every methodology fabricating flexible MSCs [61,68,73,76]. Copyright (2017), Science direct. Copyright (2013), Nature Communications. Copyright (2017), Journal of Materials Chemistry. (**b**–**d**) Mechanical and electrochemical performance of MSCs fabricated by the laser-scribing method [68]. Copyright (2013), Nature Communications.

Liu et al., developed carbon-type flexible all-solid-state MSCs using a mask-free plasma etching method, the schematic illustration is given in Figure 6a. CNT electrode and PVA/H_3_PO_4_ gel polymer electrolyte were used. They compared interdigitated SCs and conventional sandwich SCs using the same electrodes with the former exhibiting higher capacitance [68]. The power and energy densities could be handled easily by changing the dimension of interdigitated electrodes per unit area. The MSC with 12 electrodes demonstrated a capacitance of 2.02 F/cm^3^ (scan rate = 10 mV/s). The capacitance stayed at 94.1% after 6000 GCD cycles and showed stable performance with capacitance retention of 98.2% over 600 bending cycles [61]. El-Kady et al., fabricated MSCs on flexible substrates using laser-scribed graphite oxide (LSG) electrodes, which are very deformable and can be twisted and bent without influencing the structural integrity of the device. The laser scribing process utilizes changes in electrical properties, optical properties, and film structure. Figure 6b shows the mechanical performance of the LSG-MSC under strain and Figure 6c shows the CV characteristics of an MSC for various bending and twisting conditions at 1000 mV/s with the data demonstrating electrochemical stability and excellent mechanical stability irrespective of the degree of bending or twisting. As shown in Figure 6d, the flexibility and stability of the device was examined during bending and twisted state and the capacitance was reversible and the initial capacitance was sustained at 97% after 2000 cycles [68].

The studies mentioned above featured electrodes using carbon based-materials, which form an electrical double layer (EDL) at the surface. They are commonly used as active materials for SCs because of their good electric conductivity and large surface areas. Surface area is an important indicator for the performance of SCs, as ions are stored only at the surface by adsorption and desorption. Metal oxides are also outstanding candidates as they store ions over redox reactions on the surface. However, most pseudocapacitive materials do not have a large specific area nor satisfactory electrical conductivity, both of which are necessary. In this regard, Lee et al., increased the surface area using laser processing and fabricated flexible MSCs [76]. Laser-induced sintering of the metal oxide precursors allows fabricating considerably porous electrodes, forming incomplete crystal growth and agglomeration. They used a polyimide (PI) film as the flexible substrate, silver conductor, PVA as gel electrolyte and MnO_2_ and Fe_2_O_3_ as active materials. They maximized the operating voltage and achieved high volumetric energy density by using an asymmetrical configuration of hetero-pseudocapacitive metal oxides, namely, MnO_2_ and Fe_2_O_3_. By controlling the laser scan rate, the porosity of silver and metal oxides could be managed. As the scan rate increased, the size of pores decreased and simultaneously the number of pores per unit area increased. The measured CV and GCD of both electrodes showed high stack capacitance values of 160.5 F/cm^3^ (Fe_2_O_3_) and 136 F/cm^3^ (MnO_2_). Furthermore, the CV curve was continuously maintained under different bending angles (0°, 45°, 90°, and 120°), showing good deformability of the device [76].

Industrial-scale applications demand rapid production of large-area interdigitated electrodes at a low cost. In this regard, the gravure printing method is a promising option, as it provides high-speed, roll-to-roll deposition of materials at high resolution (<30 µm) [77,78]. Zhang et al., fabricated interdigitated MSCs by gravure printing on a PI substrate with Ag electrodes, graphene active material and PVA-H_2_SO_4_ solid electrolyte [73]. The gravure-printed MSCs exhibited a high capacitance value of 6.65 mF/cm^2^, high power density (0.35 mWh/cm^3^ at 300 mW/cm^3^) and high energy density (1.41 mWh/cm^3^ at 25 mW/cm^3^). The CV performance was estimated for various bending states and no considerable deviation was observed at 150 µA/cm^2^, indicating excellent flexibility [73]. Yun et al., fabricated foldable asymmetric SCs (AMSCs) by patterning graphene/ZnO nanoparticles using a resistive-type UV sensor method [79]. An organic solvent-based gel-type electrolyte containing Li^+^ ions was used for pseudo-capacitance. In each folding state, the Galinstan interconnect between adjacent separated AMSCs was completely folded. The calculated capacitance after 100 folding/releasing cycles indicated significantly stable performance. The array also exhibited mechanical stability without pronounced electrochemical degradation after 2000 folding cycles [79].

## 3. Conclusions and Future Perspectives

Realizing state-of-the-art flexible electronic devices of the future is critically dependent on flexible energy storage systems. Fabrication of devices with intrinsically flexible materials or developing effective structural configurations is crucial to impart the needed extreme deformability of the flexible SCs. Here, we have summarized some relevant fabrication methodologies and reviewed them by category. In spite of the tremendous efforts by the research community thus far, many challenges still remain, and the following issues deserve attention. Improvement of the electrolyte performance is highly stressed, as it affects capacitance, operation voltage and energy/power density. Liquid electrolytes have leakage problems, while gel-type polymer electrolytes do not have sufficient ion conductivity. In addition, the operation voltage and temperature range must be expanded for stable performance under various situations by developing organic-based electrolytes with nontoxic materials or adding electrolyte additives.

There is a limit to improving the electrochemical performance of the flexible SCs by optimizing only the active materials. Reducing the ratio of the electrochemically inactive components in electrodes is also necessary. If possible, flexible electrodes comprised of all-electrochemically-active materials must be achieved. The development of a proper passivation layer for the fabricated flexible SCs cannot be overlooked [80]. Exposure to ambient air results in drying of electrolytes and cracking and performance degradation of electrodes. A printable passivation or protective layer or cover is preferable to conventional packaging or rigid covers. The passivation layer must be easy to apply in various device sizes while maintaining mechanical and electrochemical stability.

The criteria for testing and reporting the mechanical strength and flexibility of flexible SCs must be established. The word “flexible” has two implications in the application context: bendable and stretchable. However, many bendable SCs may not be able to expand and contract. In addition, the electrochemical characteristics of flexible SCs have been tested at various bending angles, but the number of bending cycles the device can withstand is unclear in many studies. Establishing standard guidelines for reporting the electrochemical performance of flexible SCs is urgently needed, as it is difficult to compare the results across material and fabrication systems [43]. The value of capacitance is often expressed normalized to the mass of active materials, while the total mass of electrodes is used occasionally and common in commercial product literature. Restricting to only active material mass is fully understandable in academic studies since research studies only focus on preparation and proper use of materials. Total mass is relevant only to commercial products as numerous details and designs go into obtaining a final optimized product which is the goal in industry but not in academic studies; it is also beyond the scope and capability of academic labs to optimize the entire design of an SC and thus, considering total mass for normalization in the research literature may be meaningless as well as misleading.

The state-of-the-art printed devices in general, including printed supercapacitors here, do not yet match the performance of their conventional rigid counterparts in the relevant metrics. Flexibility is understandably accompanied by compromise in performance, which may be due to the choice of substrates and other active materials, theoretical limits in performance, if any, while accommodating flexibility, current limitations of printing technologies, lack of well-developed equipment, lack of standards and quality control and many others. Further advances in all these areas may help to close the gap in performance between flexible devices and their conventional rigid counterparts.

Finally, manufacturing flexible SCs on a large-scale with high throughput requires further advances, as energy storage devices are a commodity item in a consumer market, and thus, are price-sensitive. Many of the printing methods are low-cost alternatives to cleanroom-based micro and nanofabrication, which are not needed here. Reliable processing techniques and the choice of the correct materials become important in establishing a low-cost structure. As has been pointed out before, performance is not the only, or the most important, criterion in material selection, but performance to price ratio must be considered instead [81]. For example, Ru is nearly 500 times more expensive than Mn and therefore not recommended unless a RuO_2_ based pseudocapacitor shows 500-fold or greater improvement over Mn-oxide devices in all or most critical performance metrics [81]. Safety must also be a primary criterion when making choices for electrolytes and other active components of the flexible SC.
